# Supplementary material for: HIVprotI: an integrated web based platform for prediction and design of HIV proteins inhibitors
Source: J Cheminform. 2018 Mar 9;10:12. doi: 10.1186/s13321-018-0266-y (PMC5845081; doi:10.1186/s13321-018-0266-y)
Supplement: Supplementary file 2 — Additional file 2. Source code of HIVProtI web server. [file 13321_2018_266_MOESM2_ESM.zip › HIVprotI_Source-code/hivprotI/weka-3-6-6/doc/weka/core/converters/CSVLoader.html]

CSVLoader


---


|  |  |  |
| --- | --- | --- |
| **Overview** \| **Package** \| **Class** \| **Tree** \| **Deprecated** \| **Index** \| **Help** \| **Weka's home** | |  |
| **PREV CLASS**   **NEXT CLASS** | **FRAMES**    **NO FRAMES**     **All Classes** |  |
| SUMMARY: NESTED \| FIELD \| CONSTR \| METHOD | DETAIL: FIELD \| CONSTR \| METHOD |  |


---


## weka.core.converters Class CSVLoader

```
java.lang.Object
  weka.core.converters.AbstractLoader
      weka.core.converters.AbstractFileLoader
          weka.core.converters.CSVLoader
```

**All Implemented Interfaces:**: java.io.Serializable, BatchConverter, FileSourcedConverter, Loader, EnvironmentHandler, OptionHandler, RevisionHandler

---

``` public class CSVLoader extends AbstractFileLoader implements BatchConverter, OptionHandler ```

Reads a source that is in comma separated or tab separated format. Assumes that the first row in the file determines the number of and names of the attributes.


Valid options are:

```
 -N <range>
  The range of attributes to force type to be NOMINAL.
  'first' and 'last' are accepted as well.
  Examples: "first-last", "1,4,5-27,50-last"
  (default: -none-)
```

```
 -S <range>
  The range of attribute to force type to be STRING.
  'first' and 'last' are accepted as well.
  Examples: "first-last", "1,4,5-27,50-last"
  (default: -none-)
```

```
 -D <range>
  The range of attribute to force type to be DATE.
  'first' and 'last' are accepted as well.
  Examples: "first-last", "1,4,5-27,50-last"
  (default: -none-)
```

```
 -format <date format>
  The date formatting string to use to parse date values.
  (default: "yyyy-MM-dd'T'HH:mm:ss")
```

```
 -M <str>
  The string representing a missing value.
  (default: ?)
```

**Version:**
:   $Revision: 7431 $

**Author:**
:   Mark Hall (mhall@cs.waikato.ac.nz)

**See Also:**: `Loader`, Serialized Form

---

| **Field Summary** | |
| --- | --- |
| `static java.lang.String` | `FILE_EXTENSION`             the file extension. |

**Fields inherited from class weka.core.converters.AbstractFileLoader**

`FILE_EXTENSION_COMPRESSED`

**Fields inherited from interface weka.core.converters.Loader**

`BATCH, INCREMENTAL, NONE`


**Constructor Summary**

`CSVLoader()`
  
          default constructor.


| **Method Summary** | |
| --- | --- |
| `java.lang.String` | `dateAttributesTipText()`             Returns the tip text for this property. |
| `java.lang.String` | `dateFormatTipText()`             Returns the tip text for this property. |
| `Instances` | `getDataSet()`             Return the full data set. |
| `java.lang.String` | `getDateAttributes()`             Returns the current attribute range to be forced to type date. |
| `java.lang.String` | `getDateFormat()`             Get the format to use for parsing date values. |
| `java.lang.String` | `getFileDescription()`             Returns a description of the file type. |
| `java.lang.String` | `getFileExtension()`             Get the file extension used for arff files. |
| `java.lang.String[]` | `getFileExtensions()`             Gets all the file extensions used for this type of file. |
| `java.lang.String` | `getMissingValue()`             Returns the current placeholder for missing values. |
| `Instance` | `getNextInstance(Instances structure)`             CSVLoader is unable to process a data set incrementally. |
| `java.lang.String` | `getNominalAttributes()`             Returns the current attribute range to be forced to type nominal. |
| `java.lang.String[]` | `getOptions()`             Gets the current settings of the Classifier. |
| `java.lang.String` | `getRevision()`             Returns the revision string. |
| `java.lang.String` | `getStringAttributes()`             Returns the current attribute range to be forced to type string. |
| `Instances` | `getStructure()`             Determines and returns (if possible) the structure (internally the header) of the data set as an empty set of instances. |
| `java.lang.String` | `globalInfo()`             Returns a string describing this attribute evaluator. |
| `java.util.Enumeration` | `listOptions()`             Returns an enumeration describing the available options. |
| `static void` | `main(java.lang.String[] args)`             Main method. |
| `java.lang.String` | `missingValueTipText()`             Returns the tip text for this property. |
| `java.lang.String` | `nominalAttributesTipText()`             Returns the tip text for this property. |
| `void` | `reset()`             Resets the Loader ready to read a new data set or the same data set again. |
| `void` | `setDateAttributes(java.lang.String value)`             Set the attribute range to be forced to type date. |
| `void` | `setDateFormat(java.lang.String value)`             Set the format to use for parsing date values. |
| `void` | `setMissingValue(java.lang.String value)`             Sets the placeholder for missing values. |
| `void` | `setNominalAttributes(java.lang.String value)`             Sets the attribute range to be forced to type nominal. |
| `void` | `setOptions(java.lang.String[] options)`             Parses a given list of options. |
| `void` | `setSource(java.io.File file)`             Resets the Loader object and sets the source of the data set to be the supplied File object. |
| `void` | `setSource(java.io.InputStream input)`             Resets the Loader object and sets the source of the data set to be the supplied Stream object. |
| `void` | `setStringAttributes(java.lang.String value)`             Sets the attribute range to be forced to type string. |
| `java.lang.String` | `stringAttributesTipText()`             Returns the tip text for this property. |

**Methods inherited from class weka.core.converters.AbstractFileLoader**

`getUseRelativePath, retrieveFile, runFileLoader, setEnvironment, setFile, setUseRelativePath, useRelativePathTipText`

**Methods inherited from class weka.core.converters.AbstractLoader**

`setRetrieval`

**Methods inherited from class java.lang.Object**

`equals, getClass, hashCode, notify, notifyAll, toString, wait, wait, wait`

**Field Detail**

### FILE\_EXTENSION

```
public static java.lang.String FILE_EXTENSION
```

:   the file extension.


**Constructor Detail**

### CSVLoader

```
public CSVLoader()
```

:   default constructor.


**Method Detail**

### getFileExtension

```
public java.lang.String getFileExtension()
```

:   Get the file extension used for arff files.

    :   **Specified by:**: `getFileExtension` in interface `FileSourcedConverter`
    :   **Returns:**: the file extension

---


### getFileDescription

```
public java.lang.String getFileDescription()
```

:   Returns a description of the file type.

    :   **Specified by:**: `getFileDescription` in interface `FileSourcedConverter`
    :   **Returns:**: a short file description

---


### getFileExtensions

```
public java.lang.String[] getFileExtensions()
```

:   Gets all the file extensions used for this type of file.

    :   **Specified by:**: `getFileExtensions` in interface `FileSourcedConverter`
    :   **Returns:**: the file extensions

---


### globalInfo

```
public java.lang.String globalInfo()
```

:   Returns a string describing this attribute evaluator.

    :   **Returns:**: a description of the evaluator suitable for displaying in the explorer/experimenter gui

---


### listOptions

```
public java.util.Enumeration listOptions()
```

:   Returns an enumeration describing the available options.

    :   **Specified by:**: `listOptions` in interface `OptionHandler`
    :   **Returns:**: an enumeration of all the available options.

---


### setOptions

```
public void setOptions(java.lang.String[] options)
                throws java.lang.Exception
```

:   Parses a given list of options.


    Valid options are:

    ```
     -N <range>
      The range of attributes to force type to be NOMINAL.
      'first' and 'last' are accepted as well.
      Examples: "first-last", "1,4,5-27,50-last"
      (default: -none-)
    ```

    ```
     -S <range>
      The range of attribute to force type to be STRING.
      'first' and 'last' are accepted as well.
      Examples: "first-last", "1,4,5-27,50-last"
      (default: -none-)
    ```

    ```
     -D <range>
      The range of attribute to force type to be DATE.
      'first' and 'last' are accepted as well.
      Examples: "first-last", "1,4,5-27,50-last"
      (default: -none-)
    ```

    ```
     -format <date format>
      The date formatting string to use to parse date values.
      (default: "yyyy-MM-dd'T'HH:mm:ss")
    ```

    ```
     -M <str>
      The string representing a missing value.
      (default: ?)
    ```

    :   **Specified by:**: `setOptions` in interface `OptionHandler`
    :   **Parameters:**: `options` - the list of options as an array of strings **Throws:**: `java.lang.Exception` - if an option is not supported

---


### getOptions

```
public java.lang.String[] getOptions()
```

:   Gets the current settings of the Classifier.

    :   **Specified by:**: `getOptions` in interface `OptionHandler`
    :   **Returns:**: an array of strings suitable for passing to setOptions

---


### setNominalAttributes

```
public void setNominalAttributes(java.lang.String value)
```

:   Sets the attribute range to be forced to type nominal.

    :   **Parameters:**: `value` - the range

---


### getNominalAttributes

```
public java.lang.String getNominalAttributes()
```

:   Returns the current attribute range to be forced to type nominal.

    :   **Returns:**: the range

---


### nominalAttributesTipText

```
public java.lang.String nominalAttributesTipText()
```

:   Returns the tip text for this property.

    :   **Returns:**: tip text for this property suitable for displaying in the explorer/experimenter gui

---


### setStringAttributes

```
public void setStringAttributes(java.lang.String value)
```

:   Sets the attribute range to be forced to type string.

    :   **Parameters:**: `value` - the range

---


### getStringAttributes

```
public java.lang.String getStringAttributes()
```

:   Returns the current attribute range to be forced to type string.

    :   **Returns:**: the range

---


### stringAttributesTipText

```
public java.lang.String stringAttributesTipText()
```

:   Returns the tip text for this property.

    :   **Returns:**: tip text for this property suitable for displaying in the explorer/experimenter gui

---


### setDateAttributes

```
public void setDateAttributes(java.lang.String value)
```

:   Set the attribute range to be forced to type date.

    :   **Parameters:**: `value` - the range

---


### getDateAttributes

```
public java.lang.String getDateAttributes()
```

:   Returns the current attribute range to be forced to type date.

    :   **Returns:**: the range.

---


### dateAttributesTipText

```
public java.lang.String dateAttributesTipText()
```

:   Returns the tip text for this property.

    :   **Returns:**: tip text for this property suitable for displaying in the explorer/experimenter gui

---


### setDateFormat

```
public void setDateFormat(java.lang.String value)
```

:   Set the format to use for parsing date values.

    :   **Parameters:**: `value` - the format to use.

---


### getDateFormat

```
public java.lang.String getDateFormat()
```

:   Get the format to use for parsing date values.

    :   **Returns:**: the format to use for parsing date values.

---


### dateFormatTipText

```
public java.lang.String dateFormatTipText()
```

:   Returns the tip text for this property.

    :   **Returns:**: tip text for this property suitable for displaying in the explorer/experimenter gui

---


### setMissingValue

```
public void setMissingValue(java.lang.String value)
```

:   Sets the placeholder for missing values.

    :   **Parameters:**: `value` - the placeholder

---


### getMissingValue

```
public java.lang.String getMissingValue()
```

:   Returns the current placeholder for missing values.

    :   **Returns:**: the placeholder

---


### missingValueTipText

```
public java.lang.String missingValueTipText()
```

:   Returns the tip text for this property.

    :   **Returns:**: tip text for this property suitable for displaying in the explorer/experimenter gui

---


### setSource

```
public void setSource(java.io.InputStream input)
               throws java.io.IOException
```

:   Resets the Loader object and sets the source of the data set to be
    the supplied Stream object.

    :   **Specified by:**: `setSource` in interface `Loader` **Overrides:**: `setSource` in class `AbstractLoader`
    :   **Parameters:**: `input` - the input stream **Throws:**: `java.io.IOException` - if an error occurs

---


### setSource

```
public void setSource(java.io.File file)
               throws java.io.IOException
```

:   Resets the Loader object and sets the source of the data set to be
    the supplied File object.

    :   **Specified by:**: `setSource` in interface `Loader` **Overrides:**: `setSource` in class `AbstractFileLoader`
    :   **Parameters:**: `file` - the source file. **Throws:**: `java.io.IOException` - if an error occurs

---


### getStructure

```
public Instances getStructure()
                       throws java.io.IOException
```

:   Determines and returns (if possible) the structure (internally the
    header) of the data set as an empty set of instances.

    :   **Specified by:**: `getStructure` in interface `Loader` **Specified by:**: `getStructure` in class `AbstractLoader`
    :   **Returns:**: the structure of the data set as an empty set of Instances **Throws:**: `java.io.IOException` - if an error occurs

---


### getDataSet

```
public Instances getDataSet()
                     throws java.io.IOException
```

:   Return the full data set. If the structure hasn't yet been determined
    by a call to getStructure then method should do so before processing
    the rest of the data set.

    :   **Specified by:**: `getDataSet` in interface `Loader` **Specified by:**: `getDataSet` in class `AbstractLoader`
    :   **Returns:**: the structure of the data set as an empty set of Instances **Throws:**: `java.io.IOException` - if there is no source or parsing fails

---


### getNextInstance

```
public Instance getNextInstance(Instances structure)
                         throws java.io.IOException
```

:   CSVLoader is unable to process a data set incrementally.

    :   **Specified by:**: `getNextInstance` in interface `Loader` **Specified by:**: `getNextInstance` in class `AbstractLoader`
    :   **Parameters:**: `structure` - ignored **Returns:**: never returns without throwing an exception **Throws:**: `java.io.IOException` - always. CSVLoader is unable to process a data set incrementally.

---


### reset

```
public void reset()
           throws java.io.IOException
```

:   Resets the Loader ready to read a new data set or the
    same data set again.

    :   **Specified by:**: `reset` in interface `Loader` **Overrides:**: `reset` in class `AbstractFileLoader`
    :   **Throws:**: `java.io.IOException` - if something goes wrong

---


### getRevision

```
public java.lang.String getRevision()
```

:   Returns the revision string.

    :   **Specified by:**: `getRevision` in interface `RevisionHandler`
    :   **Returns:**: the revision

---


### main

```
public static void main(java.lang.String[] args)
```

:   Main method.

    :   **Parameters:**: `args` - should contain the name of an input file.


---


|  |  |  |
| --- | --- | --- |
| **Overview** \| **Package** \| **Class** \| **Tree** \| **Deprecated** \| **Index** \| **Help** \| **Weka's home** | |  |
| **PREV CLASS**   **NEXT CLASS** | **FRAMES**    **NO FRAMES**     **All Classes** |  |
| SUMMARY: NESTED \| FIELD \| CONSTR \| METHOD | DETAIL: FIELD \| CONSTR \| METHOD |  |


---
